# Endocytosis and Exocytosis in *Leishmania amazonensis* Are Modulated by Bromoenol Lactone

**DOI:** 10.3389/fcimb.2020.00039

**Published:** 2020-02-07

**Authors:** Anne C. S. Fernandes, Deivid C. Soares, Roberta F. C. Neves, Carolina M. Koeller, Norton Heise, Camila M. Adade, Susana Frases, José R. Meyer-Fernandes, Elvira M. Saraiva, Thaïs Souto-Padrón

**Affiliations:** ^1^Centro de Ciências da Saúde, Instituto de Microbiologia Paulo de Góes, Universidade Federal Do Rio de Janeiro, Rio de Janeiro, Brazil; ^2^Instituto Nacional de Ciência e Tecnologia de Biologia Estrutural e Bioimagem, Universidade Federal Do Rio de Janeiro, Rio de Janeiro, Brazil; ^3^Instituto de Biofísica Carlos Chagas Filho, Universidade Federal Do Rio de Janeiro, Rio de Janeiro, Brazil; ^4^Laboratório de Ultraestrutura Celular Hertha Meyer, Instituto de Biofísica Carlos Chagas Filho, Universidade Federal Do Rio de Janeiro, Rio de Janeiro, Brazil; ^5^Centro de Ciências da Saúde, Instituto de Bioquímica Médica Leopoldo de Meis, Universidade Federal Do Rio de Janeiro, Rio de Janeiro, Brazil

**Keywords:** *Leishmania amazonensis*, bromoenol lactone (BEL), endocytic pathways, exocytic pathways, ultrastructure, calcium-independent phospholipase A_2_

## Abstract

In the protozoan pathogen *Leishmania*, endocytosis, and exocytosis occur mainly in the small area of the flagellar pocket membrane, which makes this parasite an interesting model of strikingly polarized internalization and secretion. Moreover, little is known about vesicle recognition and fusion mechanisms, which are essential for both endo/exocytosis in this parasite. In other cell types, vesicle fusion events require the activity of phospholipase A_2_ (PLA_2_), including Ca^2+^-independent iPLA_2_ and soluble, Ca^2+^-dependent sPLA_2_. Here, we studied the role of bromoenol lactone (BEL) inhibition of endo/exocytosis in promastigotes of *Leishmania amazonensis*. PLA_2_ activities were assayed in intact parasites, in whole conditioned media, and in soluble and extracellular vesicles (EVs) conditioned media fractions. BEL did not affect the viability of promastigotes, but reduced the differentiation into metacyclic forms. Intact parasites and EVs had BEL-sensitive iPLA_2_ activity. BEL treatment reduced total EVs secretion, as evidenced by reduced total protein concentration, as well as its size distribution and vesicles in the flagellar pocket of treated parasites as observed by TEM. Membrane proteins, such as acid phosphatases and GP63, became concentrated in the cytoplasm, mainly in multivesicular tubules of the endocytic pathway. BEL also prevented the endocytosis of BSA, transferrin and ConA, with the accumulation of these markers in the flagellar pocket. These results suggested that the activity inhibited by BEL, which is one of the irreversible inhibitors of iPLA2, is required for both endocytosis and exocytosis in promastigotes of *L. amazonensis*.

## Introduction

*Leishmania* is a digenetic protozoan parasite belonging to the *Trypanosomatidae* family that causes a variety of human diseases collectively known as the leishmaniasis. In their developmental cycle *Leishmania* display distinct morphological and functional forms that adapt to different living conditions in their two main hosts. Parasites taken up by the phlebotomine vector with a blood meal, differentiate in the insect alimentary tract into procyclic promastigotes, which are flagellated and motile forms. After morphological and physiological modifications, in a process named metacyclogenesis, procyclics differentiate in a highly motile and infective metacyclic promastigotes, which are transmitted to the vertebrate host. Metacyclogenesis includes changes in gene expression, morphology, cell surface molecules expression, and in the type and amount of secreted/exocytosed molecules as well as, on the traffic of molecules in the compartments of parasite's endocytic/exocytic pathways (McConville et al., 2002; Morgan et al., 2002a,b; Besteiro et al., 2006; Lambertz et al., 2012).

As described for mammalian cells, exocytosis in *Leishmania* is mediated by both classical N-terminal secretion signal peptide and non-classical mechanism characterized by the secretion of extracellular vesicles (EVs) that accounts for at least 52% of the total protein secreted by the parasite (Silverman et al., 2008, 2010).

The total amount of secreted/excreted proteins by a cell is called exoproteome (a mixture of secreted proteins as well as those released in microvesicles and exosomes) and, in various parasites, has a different composition according to the parasite developmental form under consideration.

The use of trypanosomatids in intracellular traffic studies is particularly interesting because some developmental forms have a high rate of endocytosis and exocytosis, which occur mainly in a small area of the cell surface, the flagellar pocket membrane, whose dynamics regulates tightly the exposure of a variety of molecules on the cell surface (Naderer et al., 2004). Interestingly, endocytic and exocytic pathway organelles in these protozoa have structural and functional characteristics distinct from those of the corresponding compartments in mammalian cells (De Souza et al., 2009).

Protein trafficking along the endocytic and exocytic pathways requires membrane fusion events, which are dependent on membrane bilayer curvature modifications generated by the presence of specific phospholipids and their metabolites (Brown et al., 2003; Shin et al., 2012). A variety of studies have shown that cytoplasmic phospholipases are intimately involved in the modification of membrane phospholipids favoring the membrane deformation and consequently influencing membrane-trafficking events (Brown et al., 2003). Four of the cytoplasmic phospholipases, the iPLA_1_γ and three different PLA_2_: cPLA_2_α, iPLA_2_β and platelet-activating factor acetylhydrolase Ib (PAFAH Ib) were shown to be directly associated to the Golgi complex or other compartments of the exocytic pathway regulating the Golgi structure, the retrograde trafficking from the *cis* Golgi and endoplasmic reticulum-Golgi intermediate compartment (ERGIC) to the endoplasmic reticulum, the export from the TGN and the receptor recycling from endosomes (Morikawa et al., 2009; San Pietro et al., 2009; Bechler et al., 2010, 2012; Ben-Tekaya et al., 2010; Bechler and Brown, 2013).

Phospholipase A activities have been linked to microorganism differentiation, pathogenesis, and host cell invasion but, the exact mechanism of PLA action in this context has not yet been determined (Connelly and Kierszenbaum, 1984; Saffer et al., 1989; Snijder and Dijkstra, 2000; Belaunzarán et al., 2007, 2011, 2013). Four types of PLA_2_ have been described in bacteria, fungi and protozoa: the secreted, small molecular weight sPLA_2_s, the large cytosolic Ca^2+^-dependent cPLA_2_s, the Ca^2+^-independent iPLA_2_s and platelet activating factor acetyl hydrolase (PAF-AH) (Murakami and Kudo, 2002; Köhler et al., 2006; Sitkiewicz et al., 2007; Belaunzarán et al., 2011).

Genes encoding putative PLA_2_-like proteins have been identified in the genome of several species of *Leishmania* (TriTrypDB database), including a putative and not yet cloned PAF-AH in *L. amazonensis* MHOM/BR/71973/M2269 strain (Accession Number LAMA_000796800), that shares 29% protein sequence identity with phospholipase A2 from mice (Accession Number EDL23405). Previously, we showed that inhibition with dibucaine (a iPLA_2_ inhibitor) alters Golgi architecture and reduces the rates of endocytosis, secretion, and protein recycling in *Trypanosoma cruzi* (Souto-Padrón et al., 2006), similarly to that observed in mammalian cells (Tolleshaugh et al., 1982; Hagiwara and Ozawa, 1990; Ramanadhan et al., 1993; Lennartz et al., 1997; De Figueiredo et al., 2001; Balboa et al., 2003; Fensome-Green et al., 2007). However, similar studies in *Leishmania* have not been formally addressed with the use of tools that target the function of these proteins specifically.

In the present study we report that bromoenol lactone (BEL) inhibition modulated the metacyclogenesis, the exocytosis/secretion of both tartrate sensitive and resistant acid phosphatases, the shedding of microvesicles, the intracellular localization of GP63 and transferrin receptor and the endocytosis of BSA, Concanavalin A and transferrin by promastigotes of *L. amazonensis*, suggesting a role for sensitive iPLA2.

## Materials and Methods

### Parasites

The *Leishmania amazonensis* (MHOM/BR/75/Josefa) was maintained by inoculation into the base of the tails of BALB/c mice. Axenic promastigotes were cultured in Schneider's insect medium supplemented with 10% fetal calf serum (Gibco, Brazil) at 26°C. Promastigotes used in the experiments were harvested by centrifugation (1,500 × *g* for 12 min) from 1- to 2-day-old logarithmic phase cultures, washed and counted using a hemocytometer.

### Drugs and Reagents

Bromoenol lactone (BEL), sodium tartrate, DAPI, poly-L-lysine, and all other common reagents were from Sigma–Aldrich Chemical Co (St. Louis, MO, USA). SDS-PAGE and Western blotting equipment and reagents were from Bio-Rad Laboratories (Hercules, CA, USA) and Amersham Biosciences (Piscataway, NJ, USA). Molecular weight markers LMW were from Fermentas Life Sciences (Waltham, Massachusetts, USA). The mouse polyclonal antibody against *Leishmania* GP63 was from Acris antibodies (Herford, Germany, cat. # AM01176SU-N), and the mouse monoclonal antibody against α-tubulin was from Sigma-Aldrich (cat. #: MFCD00145891). Secondary antibodies, albumin, transferrin and concancavalin A (ConA) conjugated to Alexa Fluor® 488, and the JC-1 dye, were from Molecular Probes (Molecular Probes; Eugene, OR, USA). 1-palmitoyl-2-[6-[(7-nitro-2-1,3-benzoxadiazol-4-yl)amino]caproyl]-*sn*-glycero-3-phosphocholine (6C-NBD-PC) was purchased from Avanti Polar Lipids (Alabaster, AL, USA). [3-(4,5-dimethyl-2-thiazolyl)-5-(3-carboxymethoxy-phenyl)-2-(4-sulfo- phenyl)-2*H*-tetrazolium inner salt/5-methylphenazinium methyl sulfate] (MTS/PMS) reagent (CellTiter Aqueous MTS One Solution Cell Proliferation Assay, Promega, Madison, WI, USA). Electron microscopy resin PolyBed 812 was from Polysciences (Warrington, PA, USA).

### *In vitro* Anti-proliferative and Anti-differentiation Effects

Promastigotes were grown in Schneider's insect medium supplemented with 10% fetal calf serum (Gibco, Brazil) at 26°C in the absence or in the presence of 2.5, 5, or 10 μM BEL, and then cells were counted daily for 7 days, using a hemacytometer. To analyze the effect of BEL in metacyclogenesis, parasites in the log growth phase were incubated in the presence of BEL (2.5 μM) throughout the growth period, and the percentage of metacyclic promastigotes analyzed in cell smears stained with Giemsa (1:20), in a Zeiss Axioplan II light microscope.

### Inhibition of Phospholipase A_2_ Activities

Promastigotes of *L. amazonensis* were incubated in RPMI without serum with 2.5 μM BEL according to Ackermann et al. (1995). The inhibition treatment was done for 1 h at 26°C. Stock solution of the inhibitor was prepared in dimethyl sulfoxide (DMSO), stored at −20°C, and diluted in RPMI medium without serum immediately before use. The final concentration of the diluent never exceeds 0.1%. DMSO at the same concentration used for treatment with BEL did not affect any of the experiments showed in the present study, thus, all controls shown in this study refer to parasites not exposed to either DMSO or BEL treatment.

### Cytotoxicity Assays

To assess parasite viability, control and BEL-treated promastigotes were harvested (4,000 × *g* for 15 min), washed in Hank's balanced salt solution (HBSS) and then incubated with propidium iodide (PI; 15 μg/mL), at 37°C for 15 min. PI fluorescence associated with non-viable cells was measured by flow cytometry (544/602 nm excitation/emission) on a FACSCalibur (Becton-Dickinson, Franklin Lakes, NJ, USA). A positive control for cell lysis was prepared by treating the parasites with 0.1% saponin in PBS for 40 min, at 26°C. Flow cytometry data were analyzed using the CellQuest software (Scripps Research Institute, San Diego, CA, USA).

### Viability Assay

Control and BEL-treated parasites (2.5 μM, 1 h) were washed with sterile PBS (pH 7.2) and diluted in RPMI medium without phenol red supplemented with 10 mM glucose. Then, 2 × 10^6^ cells/well were seeded in 96-well tissue culture plate and 20 μl of a solution of 2 mg/ml MTS [3-(4,5-dimethylthiazol-2-yl)-5-(3-carboxymethoxyphenyl)-2-(4-sulfophenyl)-2H-tetrazolium salt) and 0.92 mg/ml PMS (phenazine methosulfate], prepared according to the manufacturer's instructions (Promega) were added. Samples were diluted to a final volume of 100 μl/well with RPMI without phenol red, and incubated for 3 h at 26°C. The formation of a soluble formazan product was measured using a microplate spectrophotometer reader (Spectra Max Molecular Devices M2e) at 490 nm. Negative controls for MTS/PMS consisting of promastigotes fixed with 0.4% formaldehyde for 10 min at 26°C.

### Mitochondrial Membrane Potential Analysis

Mitochondrial membrane potential (ΔΨ*m*) was investigated using the JC-1 fluorochrome, as described by Macedo-Silva et al. (2011). Briefly, control an BEL-treated promastigotes (2.5 μM, 1 h) were harvested, washed in PBS pH 7.2, added to the reaction medium containing 125 mM sucrose, 65 mM KCl, 10 mM HEPES/K^+^ pH 7.2, 2 mM Pi, 1 mM MgCl_2_ and 500 μM EGTA and counted using a hemocytometer. To evaluate the ΔΨ*m*, 2.0 × 10^7^ parasites were incubated with 10 μg/mL JC-1 for 25 min in the reaction medium, and readings were made every min immediately after the start of the 25-min incubation period, using a Molecular Devices Microplate Reader (SpectraMax spectrofluorometer). As a positive control for mitochondrial membrane depolarization, parasites were incubated with 2 μM trifluoromethoxy carbonylcyanide phenylhydrazone (FCCP). The relative ΔΨ*m* value was obtained by calculating the ratio 590 nm (red)/530 nm (green).

### Secretion Assay

Control and BEL-treated promastigotes (2.5 μM, 1 h) were washed twice in HBSS, and then incubated in this solution (2 × 10^8^ parasites/mL) for 4 h at 26°C, to allow secretion to occur. Then, aliquots were centrifuged at 2,740 × g for 10 min, at room temperature, to remove intact parasites. The supernatant (containing soluble proteins and vesicles shed/secreted by the parasites) was spun at 11,000 × *g* for 30 min, at 4°C, to remove large cell debris. The resulting supernatant—referred to henceforth as the “conditioned medium”—was spun again at 4°C for 1 h at 100,000 × *g* resulting in an “extracellular vesicles fraction” (EVs) in the pellet, and a “soluble fraction” in the supernatant. EVs were washed twice in HBSS (100,000 × *g*, 1 h, 4°C). Total protein concentration in conditioned medium, in EV and in soluble fractions was determined by Bradford, using BSA as standard (Bradford, 1976). Absorbance at 595 nm was measured (after 10 min of reaction time) in a SpectraMax M2 plate reader (Molecular Devices).

### Dynamic Light Scattering (DLS)

The conditioned media samples containing the vesicles obtained from the untreated controls and BEL-treated promastigotes as described above were diluted 100-fold in Hanks' Balanced Salt Solution (HBSS) without phenol red (Thermofisher). Particles concentration determined by Bradford was 26 mg/ml for untreated control and 10 mg/ml for BEL-treated ones. The effective diameter and the polydispersity of vesicle preparations were measured at 25°C by Dynamic Light Scattering (DLS) in an Omni Particle Sizing analyzer (Brookhaven Instruments Corp., Holtsville, NY). The multimodal distributions of particle size diameter were generated by a Non-Negatively constrained Least Squares algorithm (NNLS) based on the intensity of light scattered by each particle. All vesicle samples were analyzed under the same conditions.

### Phospholipase A_2_ (PLA_2_) Activity Assay

PLA_2_ activity present in intact parasites and in conditioned medium fractions (EVs and soluble) were determined using the fluorescent substrate 6C-NBD-PC, according to Wittenauer et al. (1984). To measure PLA_2_ activity in control and BEL treated parasites, 2 × 10^8^ promastigotes were incubated in 4 ml RPMI for 3 h, 26°C. The intact parasites, the conditioned medium and its sub-fractions were obtained as described in the preceding section. Parasites were then washed twice in HBSS and suspended in 4 ml of PBS; the conditioned medium, and the soluble fraction were separated and kept at 4°C (to measure secreted PLA_2_ activity). To concentrate protein samples from conditioned medium and from the soluble fraction, 4 mL aliquots of each sample were transferred to Amicon tubes (Amicon Ultra 15, Millipore) and centrifuged at 7,500 × *g* for 15 min, until the concentrated volume was 200 μL (50 X concentrated). EV fractions (obtained as described previously) were resuspended in 200 μL PBS, and 50 μL of each sample (parasite suspensions, concentrated conditioned media, soluble, and EV fractions) were added to wells of black 96-well plates (SLP Lifescience), C6-NBD-PC was added in the dark (5 μM final), and samples were diluted to the final volume of 200 μL/well with PBS. Fluorescence was monitored at 26°C for 60 min, in a SpectraMax M2/M2e microplate (460/534 nm excitation/emission). As a negative control, samples were analyzed without the addition of the fluorogenic substrate.

### SDS-PAGE and Western Blotting

The intact cells and conditioned media from untreated control and BEL-treated parasites to be analyzed in SDS-PAGE were boiled in 60 mM Tris-HCl pH 6.8, 5% 2-mercaptoethanol, 10% glycerol and 0.01% bromophenol blue, fractionated into 10% polyacrylamide gels (Laemmli, 1970) and transferred to nitrocellulose membranes (Hybond, Amersham Biosciences) using the Bio-Rad mini vertical Trans-Blot Cell system. Membranes were blocked in 5% non-fat dried milk in TBS (50 mM Tris-HCl, 100 mM NaCl, pH 7.5) for 1 h at room temperature, washed 3 times in TBS with 0.05% Tween-20 (TBS-T; 10 min/wash), once with TBS (5 min), and then incubated (for 18 h at 4°C) with one of the following primary antibodies: purified polyclonal anti-GP63 (1:1,000; Russel and Wilhelm, 1986) or monoclonal anti-α-tubulin (1:4,000; Sigma-Aldrich) diluted in TBS with 3% BSA and 0.05% sodium azide. Membranes were washed as described above and incubated with alkaline phosphatase-conjugated anti-mouse IgG secondary antibody (KPL), diluted to 1:1,000 in TBS with 3% BSA, for 1 h at room temperature. After a final washing cycle, bound antibodies were detected with 0.03% BCIP (5-bromo-4-chloro-3-indolyl-phosphate)/0.02% NBT (nitro blue tetrazolium) reagent (Fermentas) in alkaline phosphatase buffer (100 mM Tris-HCl pH 9.5, 150 mM NaCl, 5 mM MgCl_2_, 0.05% Tween-20). The densitometry of the gel bands was analyzed by using the Gel Doc XR+ System (Bio-Rad Laboratories, CA, USA) and the Image Lab® software.

### Acid Phosphatase Activity Analysis

Control and BEL treated (2.5 μM, 1 h) parasites (2 × 10^7^ cells/mL) were centrifuged, washed and incubated in RPMI without serum for 3 h, 26°C. Then the samples were centrifuged and the pellet of intact cells and the conditioned medium (50 μL) were incubated, at 26°C for 60 min, in a reaction mixture (0.5 mL) containing 5 mM *p*-nitrophenylphosphate (*p*-NPP), in 50 mM Mes-Hepes buffer (pH 5.0). Reactions were started by the addition of cells or conditioned medium aliquots, and stopped by the addition of 1.0 mL 1 N NaOH. Cells added after interruption of the reaction were used as a blank control. For determining the concentration of the product of p-NPP hydrolysis (*p-*nitrophenol, p-NP) released, cells were centrifuged at 1,500 × *g* for 15 min, at 26°C, and the supernatants were analyzed using a SpectraMax M2/M2e spectrofluorometer (Molecular Devices), at 425 nm, using a *p*-NP curve as standard (Gomes et al., 2006).

The analysis of phosphatase inhibition was assayed as described above, but with the addition of sodium tartrate, and the secreted acid phosphatase inhibitor at 10 mM (final concentration), immediately before the addition of either parasites, or conditioned medium. Measurements of phosphatase activity in the presence of inhibitors were normalized to control measurements in the absence of inhibitors.

### Endocytosis Assay

Control and BEL-treated promastigotes (2 × 10^6^ parasites/mL) were washed 3 times in PBS, pH 7.2, and then incubated at 26°C for 1–2 h in RPMI containing one of the following fluorescent markers: 30 μg/ml BSA-Alexa Fluor 488, 25 μg/ml transferrin-Alexa Fluor-488 and 10 μg/ml Concanavalin A (ConA)-Alexa Fluor-488 (all from Molecular Probes). After incubation with fluorescent tracers, cells were analyzed by flow cytometry, in FACSCalibur flow cytometer (Becton-Dickinson, Franklin Lakes, NJ, USA) equipped with the CellQuest software (Joseph Trotter, Scripps Research Institute, San Diego, CA, USA). Also these cells were washed in PBS (pH 7.2), fixed in 4% formaldehyde (in PBS), for 30 min at 4°C, washed in PBS, and then adhered to poly-L-lysine-coated glass coverslips for 20 min, at 26°C. Coverslips were then washed twice in PBS mounted onto slides using anti-fade solution (0.2N *n*-propyl-galate in glycerol), and observed either in a ZEISS Axioplan II epifluorescent microscope, or in a Leica TCS SPE confocal microscope.

### Transmission Electron Microscopy (TEM)

Parasites were fixed with 2.5% glutaraldehyde in 0.1 M cacodylate buffer (pH 7.2) containing 5 mM calcium chloride and 2% sucrose, for 1 h at room temperature. Then samples were rinsed in 0.1 M cacodylate buffer (pH 7.2) containing 2% sucrose, post-fixed in 1% osmium tetroxide (OsO_4_) in 0.1 M cacodylate buffer (pH 7.2) with 0.8% potassium ferrocyanide and 5 mM calcium chloride (for 1 h at room temperature), dehydrated in a graded acetone series, and embedded in PolyBed 812 resin. Ultrathin sections, obtained with a Leica (Nussloch, Germany) ultramicrotome, were stained with uranyl acetate and lead citrate, and observed using a FEI Morgagni F 268 transmission electron microscope, operating at 80 kV. The observed effects were well-recurring phenomena and the differences between control and BEL-treated parasites were readily apparent.

### Cytochemical Detection of Acid Phosphatases

Promastigotes were fixed in 1% glutaraldehyde and 3.7% sucrose in 0.1 M cacodylate buffer (pH 7.2), for 10 min at 4°C. Next, cells were washed once in 0.1 M cacodylate buffer (pH 7.2) containing 3.7% sucrose, incubated for 10 min in 0.1 M sodium acetate buffer (pH 5.0) containing 5% sucrose, and then incubated for 1 h (at 37°C, and under constant agitation) in 0.1 M sodium acetate buffer (pH 5.0) containing 2 mM sodium β-glycerophosphate, 2 mM cerium chloride and 5% sucrose. Negative control cells were incubated either in reaction medium without substrate or in reaction medium containing 10 mM of the phosphatase inhibitor sodium tartrate. After that cells were washed in sodium acetate (pH 5.0) and sodium cacodylate (pH 7.2) buffers, fixed again for 1 h in 2.5% glutaraldehyde, 3.7% sucrose and 0.1 M cacodylate buffer (26°C), and prepared for TEM (post-fixed, dehydrated, and embedded) and viewed as described above (see “Transmission Electron Microscopy”).

### Immunofluorescence

Cells were fixed in formaldehyde (4% in PBS), washed in PBS, adhered to poly-L-lysine-coated glass coverslips and washed twice in PBS. The excess of aldehyde was quenched by incubating in 50 mM ammonium chloride (in PBS), for 30 min at room temperature, and then samples were incubated in PBS containing 0.2% gelatin, 0.1% azide (“PGN” solution; Da Silva et al., 2006) for 30 min, at room temperature. Cells were permeabilized using 0.1% saponin in PGN, and then incubated with the anti-GP63 antibody (1:1,000 in PGN), for 1 h at room temperature. Then, coverslips were washed twice in PGN and incubated with Alexa Fluor 488-conjugated anti-mouse antibody (diluted to 1:400), for 1 h. Coverslips were mounted as above and observed in a ZEISS Axioplan II microscope equipped with a Color View XS camera. Negative controls were incubated with secondary antibody only (no parasite auto-fluorescence was detected).

### Statistical Analysis

Each experiment was repeated at least 3 times, and data are presented as the mean plus SEM (standard error of the mean), analyzed using the Graph-Pad-Prism 5.0 software. Comparisons between 2 groups were analyzed using Student *t* test. The differences were considered statistically significant when *p* ≤ 0.05.

## Results

### Treatment With BEL Reduces Promastigotes Differentiation Into Metacyclics, but Does Not Affect Cell Growth or Viability

**Figure 1:**
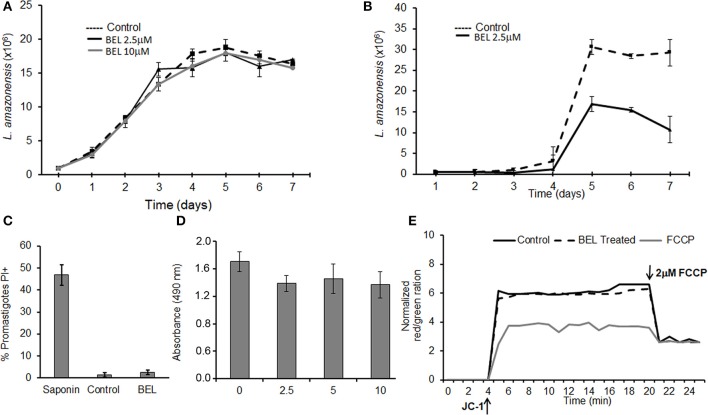
Effects of bromoenol lactone (BEL) on *Leishmania amazonensis*. **(A)** Promastigotes were grown in the absence (control) or presence of 2.5 or 10 μM BEL, and cells counted daily for 7 days, using a hemacytometer. **(B)** *L. amazonensis* metacyclogenesis, Log growth phase promastigotes were incubated with 2.5 μM BEL and the percentage of metacyclic forms were evaluated daily over 7 days of growth using a hemocytometer. **(C)** Flow cytometry analysis of untreated control and BEL-treated promastigotes (2.5 μM, 1 h, 26°C) stained with 15 μg/mL propidium iodide (PI). Saponin treated-parasites were used as a positive control. **(D)** Untreated (0) and BEL-treated promastigotes at the indicated concentrations were stained with MTS/PMS for the viability assay. **(E)** Mitochondrial membrane potential (ΔΨ*m*), in cells labeled with JC-1. The values of ΔΨ*m* were evaluated over 20 min, after which the “uncoupler” carbonyl cyanide-4-phenylhydrazone (FCCP) was added to the incubation medium, to abolish the ΔΨ*m*. The ΔΨ*m* values are expressed as the ratio of aggregate [red]/monomer [green] measured at 590/530 nm. Data represent mean ± SEM values from three independent experiments, performed in triplicates.

Previous studies using dibucaine, a non-specific inhibitor of phospholipase activity, showed the importance of iPLA_2_ for exocytic and endocytic traffic in *Trypanosoma cruzi* (Souto-Padrón et al., 2006). These results prompted us to study the role of BEL in vesicular trafficking in other human pathogens from the Trypanosomatidae family, such as *Leishmania amazonensis*. It has been demonstrated that BEL was the most active PLA_2_ inhibitor (Bordon et al., 2018), therefore we used this drug to characterize its role in some properties of promastigotes. Initially, we monitored the growth of *L. amazonensis* promastigotes for 7 days in Schneider's medium containing different concentrations of BEL (Figure 1A). Our results show that BEL, at the concentrations used, did not affect the growth of the parasites. In contrast, BEL treatment affects metacyclogenesis reducing about 65% the differentiation of promastigotes into metacyclics (Figure 1B). Importantly, parasite's membrane integrity, based on the absence of cytosolic leaky, the oxidoreductase activity and maintenance of the mitochondrial membrane potential, were not affected by BEL (Figures 1C–E). Interestingly, similar concentrations of BEL suppresses cell proliferation and leads to reduction in mitochondrial membrane potential and apoptosis induction in mammalian cells (Wilson et al., 2000; Fuentes et al., 2003; Zhang et al., 2006; Song et al., 2007; Ma et al., 2011). Based on the data described above, we chose to use 2.5 μM BEL in all the exocytosis and endocytosis experiments, since this dose did not affect parasite viability, even after more than 24 h treatment (Figure 1A).

### BEL-Sensitive PLA_2_ Activities in *L. amazonensis* Promastigotes

*Leishmania* promastigotes have a marked secretory activity, in the form of both soluble proteins and membrane-bound vesicles (Silverman et al., 2010). Thus, we assayed PLA_2_ activities in intact parasites and in “conditioned medium” (HBSS after parasite incubation for 4 h). In addition, we fractionated the conditioned medium into extracellular vesicles (EVs) and soluble fractions, and then assayed the PLA_2_ activity in these individual fractions.

**Figure 2:**
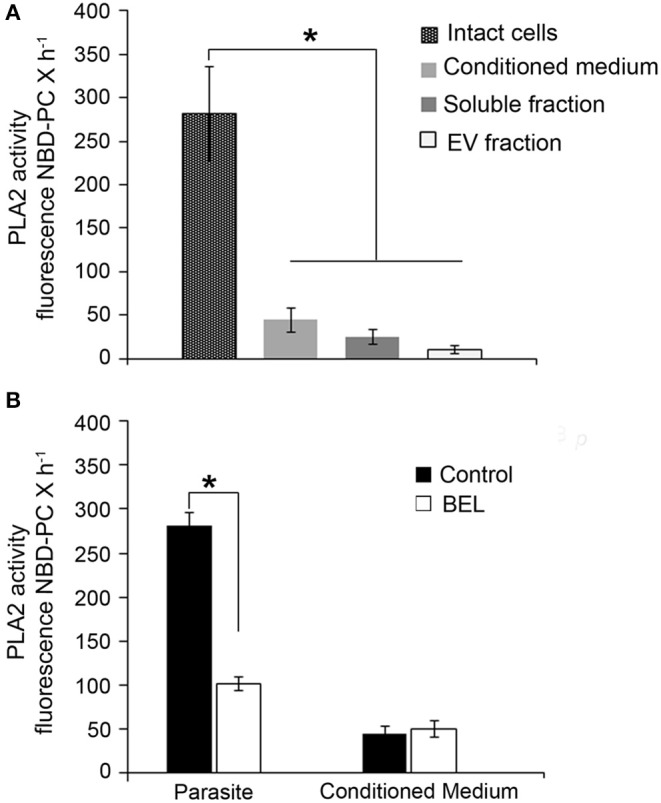
Phospholipase A_2_ (PLA_2_) activity in promastigotes of *Leishmania amazonensis*. **(A)** PLA_2_ activity was assayed in intact cells, in conditioned medium (from 2 × 10^8^ promastigotes incubated in HBSS for 4 h, 26°C), and in soluble and extracellular vesicle (EV) fractions obtained from the conditioned medium, using the fluorogenic substrate 6C-NBD-PC. **(B)** Effect of the inhibitor in the PLA_2_ activity of intact cells and conditioned media. Fluorescence measured as above was continuously monitored for 60 min 26°C in control untreated samples and in BEL treated promastigotes. Cells were treated with the inhibitor for 1 h, and then incubated for 3 h in culture medium lacking inhibitor. Data represent mean ± SEM values from three independent experiments, performed in triplicates (*p < 0.05).

Using the fluorogenic substrate 6C-NBD-PC, we detected marked PLA_2_ activity in intact promastigotes (Figure 2A). PLA_2_ activity was also detected in the conditioned medium and it was significantly higher in the soluble fraction compared with the EV fraction (Figure 2A). In order to identify if PLA_2_ activity is present in the promastigotes, we used BEL treatment in intact parasites. Our results showed that BEL-sensitive activity accounted for approximately 60% of the total PLA_2_ activity in the parasites (Figure 2B). BEL did not inhibit the phospholipase activity in the conditioned medium, suggesting no detectable iPLA_2_ activity.

### BEL Inhibits Exocytosis and Alters Golgi Morphology of Promastigotes

Inhibition of iPLA_2_ by BEL prevents exocytosis and delays protein transport between intracellular compartments from endocytic/exocytic pathways in different mammal cells such as mammary epithelial cells, mast cells, and pancreatic cells (Ramanadhan et al., 1993; Péchoux et al., 2005; Fensome-Green et al., 2007). Next we assessed whether pre-treatment of cells with BEL affected protein secretion into the conditioned medium, and which was the result of exocytosis by promastigotes.

**Figure 3:**
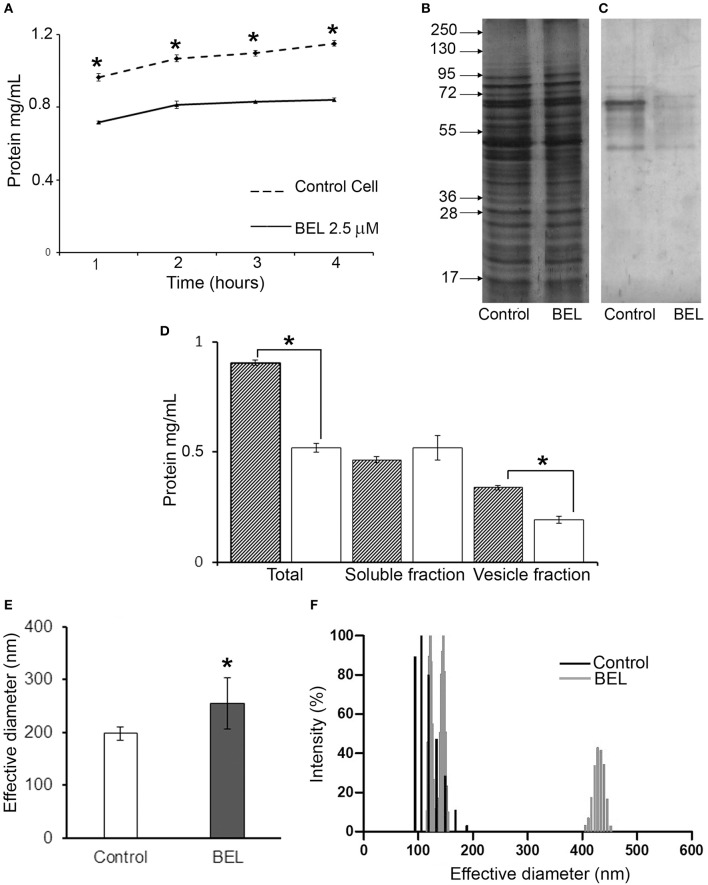
Effect of bromoenol lactone (BEL) on the protein secretion by *Leishmania amazonensis* promastigotes. **(A)** Kinetics of protein secretion by promastigotes untreated (control) or treated with 2.5 μM of BEL for 1 h, and then incubated at 26°C for 4 h in Hank's balanced salt solution (HBSS). Protein concentrations in the conditioned medium were estimated using Bradford. Data represent mean ± SEM values from three independent experiments, performed in duplicates.*p < 0.05. **(B,C)** SDS-PAGE profile of intact cells **(B)** and of conditioned media **(C)** from untreated control and BEL-treated parasites (all lanes contain samples derived from equivalent numbers of cells). Molecular weight markers are indicated on the left. **(D)** Total amount of protein in the conditioned media (Total), soluble fractions and vesicle fraction of control (gray bars) and BEL-treated parasites (white bars). **(E,F)** Effective diameter and the polydispersity of *L. amazonensis* vesicles obtained from control and BEL-treated promastigotes were analyzed by dynamic light scattering (DLS). Results shown as effective diameter in nm and % of intensity of 5 different measurements *p < 0.05.

To examine in more detail the effect of BEL on promastigote exocytosis, we measured the amount of protein secreted by promastigotes in the conditioned medium, evaluated after 1 h of incubation with BEL followed by a wash and incubation for 1–4 h in HBSS (Figure 3). Our results showed that the amount of protein secreted by control cells increased with time (Figure 3A), and this was also observed in BEL-treated parasites, but these cells secreted ~40% less protein in the conditioned medium when compared to control cells. The protein profile analyzed by SDS-PAGE depicts a decrease in the intensity of the bands between 40 and 60 kDa (Figure 3B). SDS-PAGE profiles of conditioned medium from control and BEL-treated parasites contained protein bands ranging from 45 to 95 kDa, and a reduction in the intensity of bands of 67 and 48 kDa was evidenced in the profile from BEL-treated parasites (Figure 3C). After conditioned medium fractionation, we found that the reduction in protein concentration observed after BEL treatment is related to a reduction in the vesicle fraction (~40% compared with the untreated control), since no difference was observed in the soluble fraction (Figure 3D). The size distribution evaluated by Dynamic light scattering (DLS) of the vesicles released by the promastigotes after 4 h incubation was increased after BEL treatment (Figure 3E), and contrary to the control cells, vesicles released after BEL-treatment were distributed in two populations ranging between of 100–150 and 400–450 nm of effective diameter.

**Figure 4:**
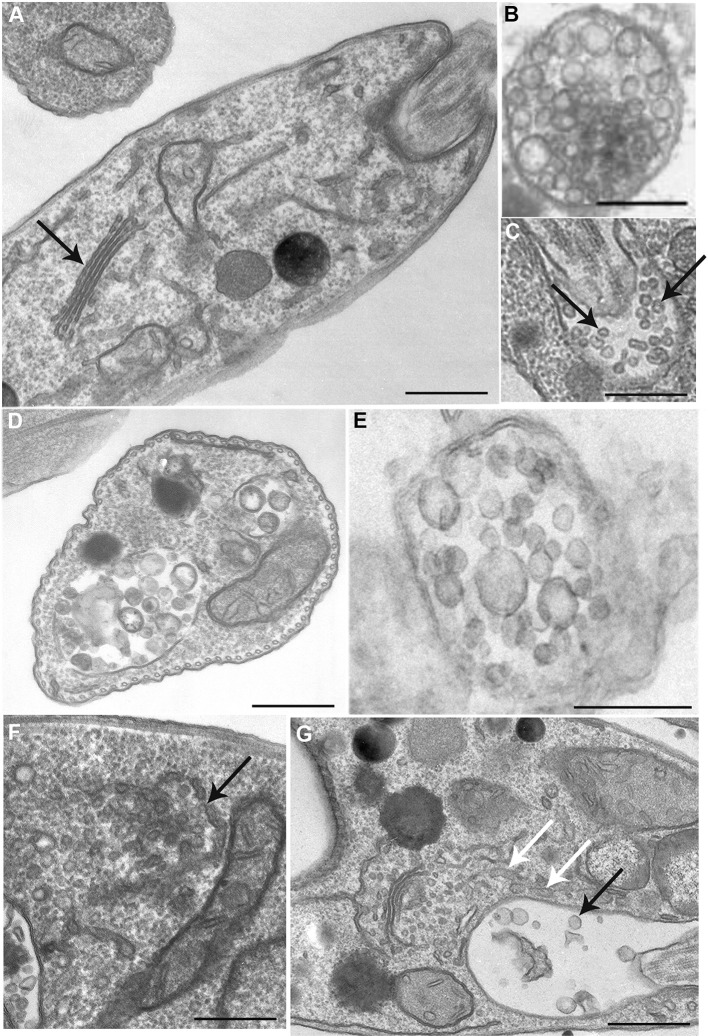
Ultrastructure of endocytic/exocytic pathway organelles in untreated-control and BEL-treated promastigotes. In control cells **(A–C)** the Golgi complex appears as a compact organelle with aligned stacks (**A**, arrow), and cells profiles contain multivesicular bodies **(B,D,E)** and numerous small vesicles within the flagellar pocket (arrows in **C**). Treatment with BEL led to intensive Golgi vesiculation (arrow in **F**), some tubules with a branched aspect in the proximity of the flagellar pocket (white arrows in **G**) and a significant decrease in the number of small vesicles in the flagellar pocket (black arrow in **G**). Representative images of three independent experiments. Bars, 300 nm.

We then analyze the ultrastructure of BEL-treated *L. amazonensis* promastigotes examining the endocytic and exocytic organelles (Figures 4A–G). As previously described in other cell types (De Figueiredo et al., 2000), BEL induced an intense Golgi and endoplasmic reticulum (ER) vesiculation in the parasite (Figures 4F,G). In some promastigotes the Golgi structure was reduced to only one or two cisterna, and the *trans*-Golgi region was filled with vesicles (Figure 4F), different from the Golgi of control cells showing preserved cisterna (Figure 4A). Aside from Golgi vesiculation, we also observed tubules with a branched aspect in the proximity of the flagellar pocket (Figure 4G, white arrows). A decrease in the number of vesicles inside the flagellar pocket was also observed after BEL treatment (Figure 4G). These data are in agreement with the reduction in the amount of protein in vesicle fraction after BEL treatment (Figures 3C,D).

### BEL Interferes With the Traffic of Secreted but Not With GPI-Anchored Glycoproteins

*Leishmania* promastigotes secrete a significant amount of glycoproteins and also have, exposed in the cell membrane, a variety of trans-membrane and GPI-anchored proteins, aside from different kinds of glycolipids (McConville et al., 2002). Endocytosis and exocytosis occur mainly in the area of the flagellar pocket membrane in the anterior region of the cell, while internalized cargo accumulates at the posterior region, in late endosomes (Ghedin et al., 2001). This strikingly polarized organization of cargo trafficking facilitates the examination of both the endocytic and the exocytic pathways in these parasites. Therefore, we examined the effects of BEL on protein trafficking in promastigotes using as models two types of cargo proteins: secreted acid phosphatases and the GPI-anchored protease GP63.

**Figure 5:**
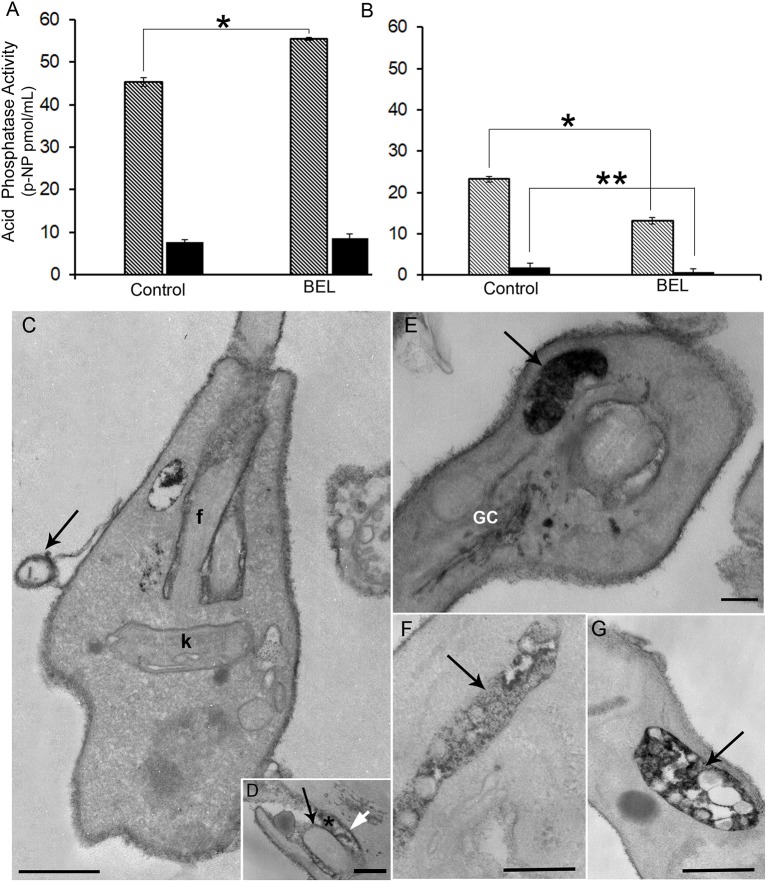
Effect of bromoenol lactone (BEL) on the acid phosphatase activity and localization in promastigotes of *Leishmania amazonensis*. Quantitative analysis of acid phosphatase activity of **(A)** promastigotes and **(B)** conditioned media assayed in the presence or absence of sodium tartrate, in intact BEL-treated and untreated cells, and in conditioned media of untreated control cells and those incubated for 1 h in the presence of BEL as described in the Methods section. Data shown as p-nitrophenyl (p-NP) released, represent mean ± SEM of three independent experiments performed in triplicates. Hatched bars = Na-tartrate sensitive and black bars = Na-resistant tartrate phosphatases. *p < 0.05; **p < 0.01. **(C–G)** Cytochemical detection of acid phosphatases in control **(C,D)** and in BEL-treated parasites **(E,G)**, using sodium β-glycerophosphate as a substrate. In control parasites, electron dense precipitate indicative of acid phosphatase activity is localized mainly in the cell body, in the flagellar membrane (black arrow in **D**), in the membrane of microvesicles (arrow in **C**), in the membrane of the flagellar pocket (white arrow in **D**) and within the flagellar pocket (asterisk in **D**). In BEL-treated promastigotes acid phosphatase activity was also observed in the Golgi complex (GC), and concentrated in multivesicular tubules (MVTs, arrows in **E,F,G**). f, flagellum; K, kinetoplast. Representative images of three independent experiments. Bars, 300 nm.

Acid phosphatase activities derive from distinct molecular entities distributed in endosomes, lysosomes, Golgi, plasma membrane, and secreted through the flagellar pocket as well (Gottlieb and Dwyer, 1981, 1982). While secreted phosphatases are sensitive to the sodium tartrate, this inhibitor does not affect the activity of acid phosphatases found on the cell surface, as integral membrane proteins (Shakarian et al., 2002, 2003). Hence, to study the effect of BEL on different acid phosphatase activities in the *L. amazonensis* promastigotes, intact cells and the conditioned medium from control and BEL treated parasites were incubated in the presence of 10 mM sodium tartrate (Figures 5A,B). Phosphatase activities detected in the promastigotes cell surface (ecto-phosphatase activities), as well as those present in cell free supernatants were mostly resistant to sodium tartrate. Treatment with BEL increased by 21% the sodium tartrate-resistant activity in the cell surface (Figure 5A). Interestingly, 43% of the sodium tartrate-resistant phosphatase activity was reduced in the conditioned medium (Figure 5B). Sodium tartrate-sensitive phosphatase activity was low in the cells and almost not detected in the secretion. Propidium iodide (PI) labeling, performed after 3 h incubation in HBSS, confirmed parasite viability, and indicated that the enzyme activity present in the conditioned medium was not due to parasites' cell damage (data not shown). These results suggest that BEL inhibited significantly the secretion/release of both acid phosphatases: the sodium tartrate-resistant and -sensitive enzymes of *L. amazonensis* promastigotes.

We also examined the distribution of phosphatase activity in promastigotes treated with BEL, by cytochemical detection in TEM. Control cells had an electron dense precipitate (corresponding to phosphatase activity) on the whole cell surface, in the flagellar pocket lumen and on the flagellum and flagellar pocket membranes (Figures 5C,D). Contrasting, treatment with BEL led to a marked decrease in the amount of precipitate in the flagellar pocket, both in its lumen and membrane (Figure 5E) although it has not interfered with the intensity of reaction observed in the cell body and flagellar membranes. On the other hand, we observed an increase in acid phosphatase reactivity in intracellular compartments, including the Golgi (Figure 5E), vesicles located near the flagellar pocket and the tubulovesicular compartment or multivesicular tubule (MVT) (Figures 5F,G).

**Figure 6:**
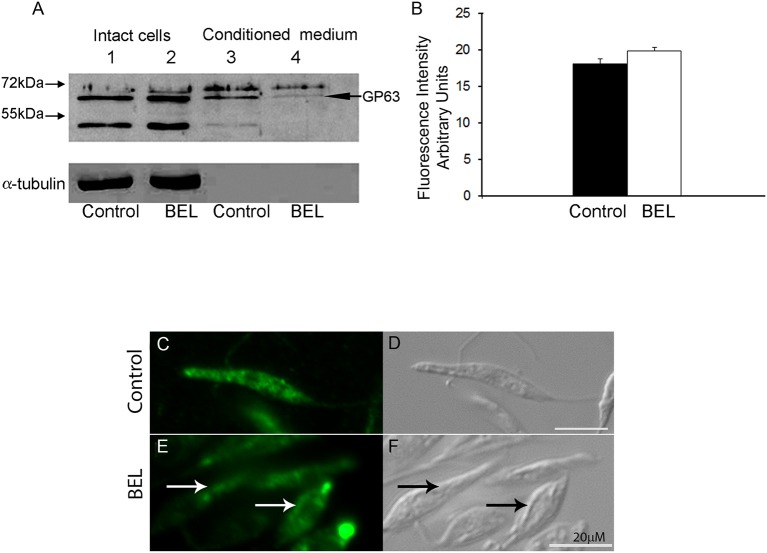
Effect of bromoenol lactone (BEL) in the intracellular localization of GP63 in *Leishmania amazonensis* promastigotes. **(A)** Immunoblots of intact cell (lanes 1 and 2) and conditioned medium (lanes 3 and 4), probed with a polyclonal anti-GP63. Lanes 1 and 3, untreated controls; lanes 2 and 4, BEL-treated parasites. Molecular weight markers are indicated on the left, and the band corresponding to GP63 is indicated on the right. α-tubulin was used as loading control. **(B)** Flow cytometry analysis of GP63 intensity fluorescence in pre-fixed and permeabilized cells showing no statistically significant accumulation of the protein in BEL treated cells. **(C–E)** Fluorescence microscopy analysis of permeabilized cells labeled with anti-GP63 antibody. In control cells **(C,D)**, GP63 labeling was observed in small organelles distributed in the cell body **(C)**. In contrast, in BEL-treated cells **(E,F)**, labeling was less intense in the cytoplasm, and was concentrated in enlarged compartments in the anterior and posterior regions of the cell body, and in elongated structures that resembled the multivesicular tubule, running along the cell body (arrows in **E** and **F**). Bars, 20 μm.

To evaluate the effect of BEL on the trafficking of a different type of surface protein, we examined the distribution of GP63 in BEL-treated parasites. GP63, the major surface protein of *Leishmania*, is a metalloprotease that occur as GPI-anchored (63 kDa) or unanchored or soluble (65 kDa) isoforms (Ellis et al., 2002; McGwire et al., 2002). Western blotting analysis of the GP63 contents in whole cell extracts of control and BEL-treated promastigotes revealed no significant difference in the intensity and in the pattern of bands (Figure 6A, lanes 1 and 2). However, in profiles of conditioned medium, we observed two major bands corresponding to the two GP63 isoforms in control samples. The total amount of GP63 was reduced after treatment with BEL (Figure 6A, lanes 3 and 4). Although we observed a reduction in the intensity in both isoforms, the 63 kDa GPI-anchored isoform was the one that suffered a larger reduction (Figure 6A, lanes 3 and 4). Flow cytometry of control and BEL-treated parasites permeabilized with saponin and labeled with anti-GP63 antibody showed that, in BEL-treated parasites, the intensity of labeling was slightly higher than that observed in the control (Figure 6B), but not statistically significant. We then asked if the increased fluorescence intensity of BEL-treated parasites was due to a reduction in GP63 secretion, with intracellular retention and/or redistribution of GP63, as observed with the phosphatase activity (Figure 5). Immunofluorescence analysis of GP63 localization showed that, in control cells, labeling was more intense (Figure 6C) than in BEL-treated cells and we observed a significant increase in the protein labeling at the posterior region of the cell body, and also in tubular compartments that ran along the body of the parasite (Figure 6E).

### BEL Reduces Endocytosis, in *L. amazonensis* Promastigotes

**Figure 7:**
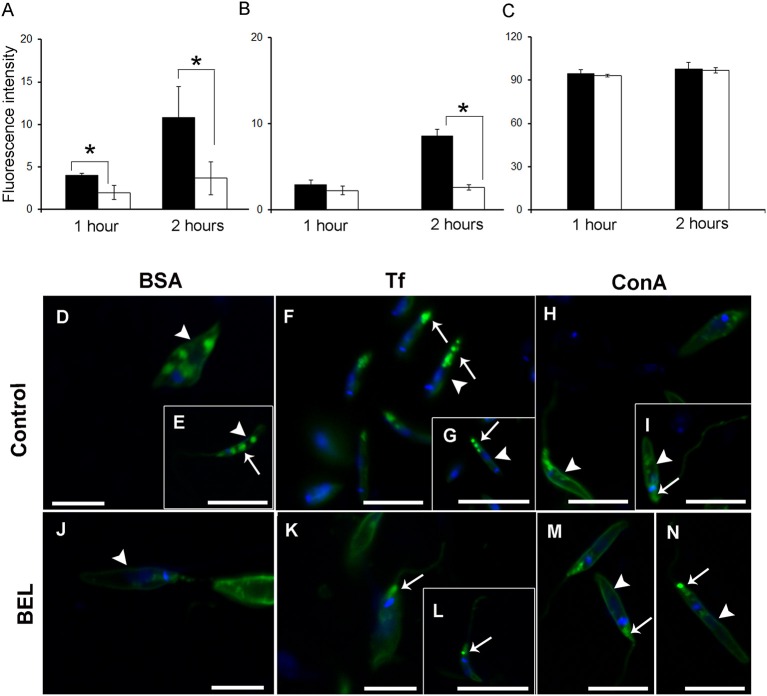
Influence of bromoenol lactone (BEL) on the endocytic activity of *Leishmania amazonensis*. Promastigotes were incubated with **(A)** bovine serum albumin (BSA), **(B)** transferrin (Tf), and **(C)** Concanavalin A (ConA) tracers and the fluorescence intensity analysis by flow cytometry. Data represent mean ± SEM of three independent experiments, performed in duplicates. *p < 0.05. **(D–I)** Untreated controls and **(J–N)** BEL-treated promastigotes were incubated with Alexa Fluor 488-labeled BSA **(D,E,J)**, Tf **(F,G,K,L)**, and ConA **(H,I,M,N)** for 2 h. In control cells, labeled BSA and Tf **(D–G)** were observed on the cell surface (arrowheads) and in cytoplasmic compartments mainly the ones at the posterior end of the cells (arrows in **E–G**). In BEL treated cells, BSA **(J)** and transferrin **(K,L)** signal occur at the cell surface (arrowhead in **J**) and at the flagellar pocket (arrows in **K,L**). Control cells incubated with fluorescent ConA had intense signal on the cell surface (arrowheads in **H,I**), and in the flagellar pocket (arrow in **I**) and this labeling pattern was not altered by treatment with BEL **(M,N)**. The DNA was stained with DAPI. Bars: 20 μM.

To study the effect of BEL on the endocytosis of promastigotes, we monitored the internalization of three fluorescent-labeled tracers: bovine serum albumin (BSA), transferrin (Tf) and ConA by flow cytometry and fluorescence microscopy, after 1 and 2 h incubation. We observed that the fluorescence intensity of control cells incubated with labeled BSA or transferrin (but not ConA) was ~3 fold higher after 2 h incubation with the tracers, when compared with 1 h incubation (Figures 7A–C). In BEL-treated parasites incubated with fluorescent BSA we observed a reduction of ~40 and 60% in the intensity of labeling compared with the control, after 1 and 2 h of incubation, respectively. BEL reduced by 70% the intensity of the transferrin labeling after 2 h, but it did not affect the labeling of promastigotes with ConA (Figures 7B,C). Moreover, we observed that in control cells, after 2 h incubation, mostly of the fluorescent BSA labeling was concentrated at the posterior end of the parasite (Figures 7D,E), compatible with tracer localization in cytoplasmic compartments of the endocytic pathway, such as late endosomes, which are located in this area (Weise et al., 2000). In BEL treated cells, BSA (Figure 7J) signal was low occurring at the cell surface and in some cytoplasmic compartments.

Control parasites incubated with Tf showed a labeling concentration in cytoplasmic compartments in the posterior region of the parasite (Figures 7F,G). Although treatment with BEL did not modify the percentage of labeled cells, compared with the control, Tf fluorescence in BEL-treated cells was small and mostly on the surface and in the flagellar pocket (Figures 7K,L), rather than at the posterior end of the cell.

ConA-labeled promastigotes depicted a quite distinct staining pattern from that found in parasites labeled with the other two tracers. In control parasites, ConA fluorescence was concentrated on the cell surface, in the flagellar pocket, and in elongated structures (resembling the multivesicular tubule described in *L. mexicana*) near the kinetoplast and the nucleus (Figures 7H,I). In most BEL-treated parasites, cell surface and flagellar pocket labeling were still apparent, but only a few cells had clear labeling of elongated cytoplasmic structures resembling the tubular compartments (Figures 7M,N).

## Discussion

Our group produced the first evidence for PLA_2_ role on endocytic and exocytic traffic in Trypanosomatids, in a study with epimastigotes of *T. cruzi* treated with dibucaine (Souto-Padrón et al., 2006). In that study, we observed effects suggestive of subversion of the regular traffic of proteases to the reservosomes; however, dibucaine is not a specific inhibitor of iPLA_2_, and it is possible that some of the effects observed were off-target.

Here, we used BEL to study its effect on endo/exocytosis and traffic of different types of membrane proteins to the cell surface, in promastigotes of *L. amazonensis*. Although BEL is an irreversible and more specific iPLA_2_ inhibitor, it has been described that BEL may also inhibit a magnesium-dependent phosphatidic acid phosphohydrolase, leading ultimately to apoptosis cell death in mammalian cells (Fuentes et al., 2003). Genes encoding putative phosphatidate phosphohydrolase proteins have been identified in the genome of several trypanosomatids (TriTrypDB database), including the *L. amazonensis* MHOM/BR/71973/M2269 strain, but none have been cloned or studied further yet. Among those, one type of phosphatidate phosphatase contains a conserved PAP-2 superfamily domain (Interpro IPR000326) and 3 putative genes coding for them were identified in the genome of *L. amazonensis* MHOM/BR/71973/M2269 strain (TriTrypDB Accession numbers LAMA 000277200, LAMA_000277300, and LAMA_000302500). The other type of magnesium-dependent phosphatidic acid phosphohydrolase identified in *L. amazonensis* (LAMA000023900) contains a Lipin (Interpro IPR007651) and Lipin/Ned1/Smp2 (Interpro IPR013209) conserved domains that strongly align with the sequences found in the yeast (NCBI AHY76617.1) and mice (NCBI NP_001186047.1) orthologs. Therefore, although not tested here, BEL might be also inhibiting another target enzyme in *Leishmania*. However, we could not found the characteristic alterations described for BEL-inhibition of magnesium-dependent phosphatidic acid phosphohydrolase (Fuentes et al., 2003) as the mitochondrial potential and apoptosis cell death in the parasites in our conditions. Moreover, treatment of *L. amazonensis* promastigotes with 2.5 μM BEL inhibited PLA_2_ activity by 60% (Figure 2B) without causing cell death, even after more than 24 h treatment (Figure 1A). In contrast, this concentration of BEL is sufficient to reduce the mitochondrial membrane potential in different types of mammalian cells (Ma et al., 2011; Nordmann et al., 2014; Rauckhost et al., 2015). Contrary to mammalian cells, BEL did not inhibit *L. amazonensis* proliferation at our conditions (Sanchez and Moreno, 2002; Balboa et al., 2003; Song et al., 2007). However, treatment with BEL significantly reduced the differentiation of promastigotes into metacyclic forms (Figure 1B). In Trypanosomatids, metacyclogenesis is an important step that occurs in the insect vector which enables parasites to survive within the vertebrate host (Avila et al., 2003; Pinto-da-Silva et al., 2005). The structural changes and expression of molecules observed during metacyclogenesis involve the activation of synthesis and degradative pathways that are dependent on the fusion of cytoplasmic compartments (Besteiro et al., 2007). We believe that changes in the fusion of these compartments caused by BEL, may participate in the reduction of metacyclogenesis rate in *L. amazonensis*.

In different pathogenic microorganisms (including bacteria and protozoa) PLA_2_ activity is important for the pathogen interaction with host cells (Connelly and Kierszenbaum, 1984; Saffer and Schwartzman, 1991; Silverman et al., 1992). In *Leishmania*, different studies showed the presence of PLA_2_ activity in *L. amazonensis* and of a platelet-activating factor acetylhydrolase (PAF-AH) in *L. major* (Henriques et al., 2003; Passero et al., 2008; Pawlowic and Zhang, 2012). Unlike that reported by Passero et al. (2008), we showed here that most PLA_2_ activity in *L. amazonensis* promastigotes is present in the parasite and not in the culture supernatant (conditioned medium) (Figure 2A). A key difference that could explain this variation was that we evaluated PLA_2_ activities in the intact parasite and not in cell lysates. Also, unlike what was shown by these authors for the inhibition of PLA_2_ activity using betamethasone, we observed that BEL inhibited significantly the iPLA_2_ activity in intact cells, but did not affect the PLA_2_ activity secreted into the conditioned medium (Figure 2B).

In mammalian cells iPLA_2_ regulates Golgi and ERGIC morphology, as well as membrane trafficking in and out of these organelles and iPLA_2_ inhibition causes intense Golgi fragmentation (Mayorga et al., 1993; De Figueiredo et al., 1998, 1999, 2000; Ben-Tekaya et al., 2010; Bechler et al., 2012). We observed similar changes in the Golgi of promastigotes after BEL treatment (Figures 4F,G). BEL also led to enlargement of the MVT and other tubular structures located in the anterior region of the parasite (Figures 4F,G). In promastigotes this region is the site of intense endo/exocytic traffic. In analogy to other cellular models, it is likely that the enlargement of endocytic pathway compartments indicates changes in vesicular traffic that lead to inhibition of both exocytosis and endocytosis (Mayorga et al., 1993; Lennartz et al., 1997; De Figueiredo et al., 1998). In line with these observations, we detected that treatment with BEL reduced around 50% the overall amount of protein in the parasites and in the vesicle fraction. Images of the flagellar pocket of control cells showed a large number of vesicles of different sizes (Figure 4C), including small vesicles (50–100 nm) likely derived from exosomes exocytosis, and larger “microvesicles” (up to 300 nm in diameter), whose membrane derives directly from the flagellar pocket membrane. Microvesicles are also observed budding from the flagellar pocket membrane at the exit of flagellar pocket, from the cell body and the flagellar membranes.

Our data are consistent with that presented by Silverman et al. (2010), showing that exosome release is a major mechanism of protein secretion by *Leishmania*. However, we also noted a clear reduction in the number of microvesicles within and outside the flagellar pocket of treated parasites. Calcium-independent PLA_2_ activity has been associated to the budding of microvesicles in lipid-rafts enriched plasma membrane (Mizuno-Kamiya et al., 2001; Nakano et al., 2009). Coincidentally, *Leishmania* plasma and flagellar membranes possess a high concentration of lipid rafts (Pimenta and De Souza, 1987) and an intense microvesicle budding activity (Saraiva et al., 1989; Vannier-Santos et al., 1992). In conclusion, our results suggest that BEL-sensitive target in *Leishmania* is also involved in controlling both the fusion of endocytic pathway compartments to the flagellar pocket membrane, which releases exosomes, and the direct budding of microvesicles from different regions of the parasite membrane. Interestingly, control parasites released microvesicles with similar size to that described by Barbosa et al. (2018). However, BEL-treated promastigotes besides the population alike the control cells, released also a second population of microvesicles, ranging from 400 to 450 nm of relative diameter.

In *Leishmania*, both acid phosphatases and GP63 are targeted to the plasma membrane, but may also be found free in the extracellular medium (Gottlieb and Dwyer, 1982; Jaffe and Dwyer, 2003). Inhibition by BEL increased the total acid phosphatase activity in promastigotes (Figure 5A), and changed the distribution of the tartrate-sensitive enzyme (Figures 5C–G). This activity, which is regularly secreted in control parasites, accumulated in MVTs after treatment with BEL, as seen in TEM images (Figures 5E–G). BEL treatment, however, did not affect the targeting of the sodium tartrate-resistant acid phosphatase to the plasma membrane, since cytochemical detection of phosphatases in BEL-treated parasites show an amount of cell surface precipitate similar to that observed in control cells (Figures 5C,E). The presence of a significant amount of tartrate-resistant phosphatase activity in the conditioned medium obtained from control parasites was in line with the ultrastructural data showing that control promastigotes released a higher amount of EVs in the conditioned medium. Its reduction after treatment with BEL is consistent with the decrease in the number of vesicles within the flagellar pocket also observed by TEM.

The GP63 is predominantly expressed on the surface of *Leishmania* promastigotes in a GPI-anchored form, associated with lipid rafts, or as an “unanchored” GP63 secreted into the extracellular medium (Weise et al., 2000; Denny et al., 2001; Ellis et al., 2002; McGwire et al., 2002). Treatment with BEL reduced the amount of GP63 released extracellularly and increased the labeling of compartments located in the posterior region of the parasite (Figure 6E), which include the MVT (Weise et al., 2000).

Anchored and unanchored GP63 isoforms are secreted by different routes that depend on the presence of GPI-anchor and N-glycosylation, respectively (Ellis et al., 2002). Our data suggest that changes in the structure of the ER and Golgi after BEL lead to changes in the glycosylation pattern of the unanchored GP63 and to their accumulation in the parasite as observed in the immunofluorescence image (Figure 6E). On the other hand GPI-anchored GP63, which targeting was previously described in *L. mexicana* (Ellis et al., 2002) as being regulated exclusively by the presence of a GPI anchor, appeared unaffected by BEL treatment as evidenced in Figures 6C,E. More substantial changes were observed in the conditioned medium after BEL treatment that reduced around 40% the amount of secreted GP63. Among the two bands observed the most significant reduction was on the 63 kDa GPI anchored GP63. The GPI-anchored GP63 may be released from the cell surface by autoproteolytic cleavage (McGwire et al., 2002). As there are no reports of BEL action inhibiting autoproteolytic activities, we believe that the reduction of the 63 kDa band observed in the conditioned medium is related to the reduction in its release through the microvesicle secretion (Silverman et al., 2010). Indeed, our results suggest that the total amount of microvesicles in the incubation medium is reduced after treatment with BEL (Figures 3G,I).

We also determined whether BEL-target inhibition would be involved in the fusion of the endocytic compartments. As previously mentioned the uptake of molecules in *Leishmania* occurs through the flagellar pocket, and both fluid-phase endocytosis (of BSA and other tracers) and receptor-mediated endocytosis—of transferrin (Voyiatzaky and Soteriadou, 1992), LDL (Bastin et al., 1996), and hemoglobin (Singh et al., 2003)—have been reported. iPLA_2_ inhibitors (such as dibucaine) decrease transferrin (Tf) endocytosis in rat muscle cells (Hagiwara and Ozawa, 1990), and BSA endocytosis in *T. cruzi* (Souto-Padrón et al., 2006). Our results using three different endocytosis tracers—BSA, Tf and Concanavalin A—showed that BEL-target inhibition is required for both fluid-phase and receptor mediated endocytosis. Nevertheless, BEL had different effects on the endocytosis of fluid-phase (BSA) and receptor-mediated (Tf) tracers by the promastigotes. Inhibition of BSA endocytosis by BEL was partial (~60%), and similar to that observed in epimastigotes of *T. cruzi* treated with dibucaine (Souto-Padrón et al., 2006). This significant (albeit not full) reduction in BSA endocytosis contrasts with a more profound effect of BEL in Tf internalization (Figures 7F,G,K,L). Bromoenol lactone did not affect surface labeling of fluorescent Tf, suggesting that its target activity is not required for Tf receptor (TfR) recycling to the cell surface, differently from that described for HeLa cells and hepatocytes (De Figueiredo et al., 2001). Our data about the TfR trafficking are in agreement with the recently described trafficking of HbR in *Leishmania* (Bahl et al., 2015), which is independent of GP63 and secretory acid phosphatase pathway. In contrast, BEL-target activity appears essential for Tf uptake, since the endocytosis of this tracer was restricted to the flagellar pocket in BEL-treated cells, and was absent from internal endocytic compartments (Figures 7F,G,K,L). Further studies are required to confirm that BEL-target has a role in TfR endocytosis, but not in receptor recycling to the surface. Unlike procyclic/bloodstream *Trypanosoma brucei* (Brickman et al., 1995), *L. amazonensis* promastigotes did not internalize large amounts of labeled ConA, which was located primarily on the surface and flagellar pocket (Figure 7). However, some parasites displayed internal ConA labeling, in elongated structures that probably correspond to tubular compartments of the endocytic pathway. Internal labeling with ConA disappeared after BEL-treatment, and this lectin was concentrated in the flagellar pocket, reinforcing the notion that BEL-target activity (presumably at the flagellar pocket membrane) is important to initiate endocytosis by vesicle budding into the cytoplasm, regardless of the endocytic route used.

In conclusion, we showed that BEL-target activity is required for three key processes in *Leishmania*, namely: the internalization of nutrients by endocytosis, the secretion of vesicles and the differentiation of promastigotes into infective metacyclic forms. Interestingly, it has been shown that although their hepatotoxicity, BEL treatment decreased parasite load in a cutaneous murine model, suggesting further chemical modifications in the molecule that could decrease this side effect maintaining the leishmanicidal activity (Bordon et al., 2018).

## Data Availability Statement

The datasets generated for this study are available on request to the corresponding author.

## Ethics Statement

The animal study was reviewed and approved by Ethics Committee for Animal Experimentation of the Health Sciences Center, Federal University of Rio de Janeiro (Protocol No. IBCCF-085).

## Author Contributions

TS-P conceived and designed the experiments. AF, DS, RN, CK, SF, NH, and CA performed the experiments. AF, DS, CK, SF, NH, CA, JM-F, ES, and TS-P analyzed the data. AF, DS, CK, NH, CA, SF, JM-F, ES, and TS-P wrote the manuscript. All authors read and approved the final version of the manuscript.

### Conflict of Interest

The authors declare that the research was conducted in the absence of any commercial or financial relationships that could be construed as a potential conflict of interest.
